# Comparative Analysis of Long Non-Coding RNA Expression and Immune Response in Mild and Severe COVID-19

**DOI:** 10.3389/fmolb.2022.835590

**Published:** 2022-04-27

**Authors:** Yongting Zhang, Fan Shi, Yuchong Wang, Yuting Meng, Qiong Zhang, Kaihang Wang, Ping Zeng, Hongyan Diao

**Affiliations:** State Key Laboratory for Diagnosis and Treatment of Infectious Diseases, National Clinical Research Center for Infectious Disease, Collaborative Innovation Center for Diagnosis and Treatment of Infectious Diseases, The First Affiliated Hospital, College of Medicine, Zhejiang University, Hangzhou, China

**Keywords:** COVID-19, lncRNA, PBMC, T-cell, monocyte

## Abstract

**Background:** Coronavirus disease 2019 (COVID-19) is a worldwide emergency, caused by severe acute respiratory syndrome coronavirus 2 (SARS-CoV-2). Long non-coding RNAs (lncRNAs) do not encode proteins but could participate in immune response.

**Methods:** In our study, 39 COVID-19 patients were enrolled. The microarray of peripheral blood mononuclear cells from healthy and COVID-19 patients was applied to identify the expression profiles of lncRNAs and mRNAs. Identified differentially expressed (DE) lncRNAs were validated by qRT-PCR. Then, the lncRNA–mRNA network was constructed and visualized using Cytoscape (3.6.1) based on the Pearson correlation coefficient. The enrichment of DE mRNAs was analyzed using Metascape. The difference in frequencies of immune cells and cytokines was detected using CIBERSORT and ImmPort based on DE mRNAs.

**Results:** All patients with COVID-19 displayed lymphopenia, especially in T cells, and hyper-inflammatory responses, including IL-6 and TNF-α. Four immune-related lncRNAs in COVID-19 were found and further validated, including AC136475.9, CATG00000032642.1, G004246, and XLOC_013290. Functional analysis enriched in downregulation of the T-cell receptor and the antigen processing and presentation as well as increased apoptotic proteins, which could lead to T-cell cytopenia. In addition, they participated in monocyte remodeling, which contributed to releasing cytokines and chemokines and then recruiting more monocytes and aggravating the clinical severity of COVID-19 patients.

**Conclusion:** Taken together, four lncRNAs were in part of immune response in COVID-19, which was involved in the T-cell cytopenia by downregulating the antigen processing and presentation, the T-cell receptor, and an increased proportion of monocytes, with a distinct change in cytokines and chemokines.

## Introduction

The coronavirus disease 2019 (COVID-19) outbreak has caused a worldwide emergency owing to its rapid spread and high mortality rate. Mostly, COVID-19 patients may have asymptomatic or mild symptoms, while severe patients can develop pneumonia to acute respiratory or multiorgan failure and death (Yang Y et al.,2020).

Multiple studies, focused on COVID-19, have highlighted the changes in peripheral immune response, including marked pro-inflammatory cytokine release and pronounced lymphopenia, especially reduction in T cells (Song et al., 2020). Therefore, there is an urgent need for further studies on the host immune response to screen prognostic and diagnostic indicators and to provide appropriate therapeutic interventions for severe COVID-19.

Long non-coding RNAs (LncRNAs) are a class of molecules with more than 200 nucleotides in length, which are incapable of coding proteins but participate in the regulation of gene expression through epigenetic, transcriptional, and post-transcriptional changes (Shen et al., 2020).

Recent studies primarily demonstrated that lncRNAs took part in various biological and physiological processes, including the cell cycle and proliferation, apoptosis, and differentiation (Wang et al., 2014). Lnc-DC was exclusively upregulated in DCs during DC differentiation, with regulation of CD40, CD80, CD86, and HLA-DR. The knockdown of lnc-DC failed to present antigens, activate T-cells, and induce cytokine production (Chen et al., 2017).

Therefore, it is essential to explore the potential of lncRNA in peripheral immune response to provide immunotherapy targets in COVID-19. In this study, we applied microarrays to investigate the potential lncRNA of peripheral blood mononuclear cells (PBMCs) from COVID-19 patients.

## Materials and Methods

### Patients

Thirty-nine COVID-19 patients and five healthy donors were enrolled, admitted in The First Affiliated Hospital, Zhejiang University School of Medicine, between 28th January and 20th February, 2020. COVID-19 was confirmed using the SARS-CoV-2-specific RT-PCR test. The patients (Supplementary Table 1) were diagnosed with the severe, who required critical care and met one or more of following criteria: dyspnea and respiratory rate ≥30 time/min, blood oxygen saturation ≤93%, PaO_2_/FiO_2_ ratio <300 mmHg, and lung infiltrates on CT scan >50% within 24–48 h, or those who exhibited respiratory failure, septic shock, and/or multiple organ dysfunction/failure. Meanwhile, all patients’ demographics, clinical characteristics, and laboratory results are shown in Tables 1, 2.

**TABLE 1:** Demographics and clinical characteristics of all patients.

Variable/Group	Normal	Mild (26)	Severe (13)	*p*-value
Age (years)	-	44.5	72	<0.05
Male (%)	-	13 (50%)	9 (70%)	NS
Onset of symptoms to hospital admission, days	-	6.5 (3–20)	9 (4–14)	<0.05
Exposure to Wuhan	-	11 (42%)	4 (31%)	NS
Any comorbidity				
Hypertension	-	7 (27%)	10 (77%)	<0.05
Diabetes	-	3 (8%)	2 (15%)	NS
Malignancy	-	0	0	
Chronic liver disease	-	0	0	
Fever				
Highest temperature (°C)				
<37.3	-	4 (15%)	3 (23%)	
37.3–38.0	-	13 (50%)	6 (46%)	
38.1–39.0	-	8 (30%)	2 (15%)	
>39.0	-	1 (5%)	2 (15%)	
Cough	-	14 (54%)	9 (69%)	NS
Expectoration	-	13 (50%)	6 (46%)	NS
Myalgia or fatigue	-	8 (31%)	1 (8%)	NS
Nausea and vomiting	-	1 (4%)	1 (8%)	NS
Sore throat	-	3 (12%)	0	NS
Shortness of breath	-	5 (20%)	2 (15%)	NS
Chest pain	-	2 (8%)	0	NS
Diarrhea	-	2 (8%)	4 (31%)	NS

Data are median or n (%), compared by the Mann–Whitney U-test or χ^2^ test between mild and severe COVID-19.

**TABLE 2:** Laboratory results of hospitalized patients with infected SARS-Cov-2.

Laboratory results	Normal range	Normal	Mild (26)	Severe (13)	*p*-value
Laboratory results	4.0–10.0*10^9	-	5.1	13	<0.05
Leukocyte (10E9/L)	2.0–7.0*10^9	-	3.3	11.9	<0.01
Neutrophil (10E9/L)	0.8–4.0*10^9	-	0.8	0.5	<0.01
Lymphocyte (10E9/L)	3.68–5.13	-	4.3	3.95	NS
Red blood cells (10E12/L)	113–151	-	130.5	121	NS
Hemoglobin (g/L)	101–320	-	197	174	<0.05
Platelet (10E9/L)	0–700	-	400	705	<0.01
D-dimer (ug/L)	0.00–20.06	-	13.23	6.58	NS
IFN-γ (pg/ml)	0.00–2.31	-	3.82	7.94	<0.05
IL-10 (pg/ml)	0.00–4.13	-	1.14	2.09	NS
IL-2 (pg/ml)	0.00–6.61	-	13.58	47.18	<0.05
IL-6 (pg/ml)	0.00–33.27	-	9.345	22.51	NS
TNF-α (pg/ml)	70.0–140.0	-	137	126	NS
Complement3 (mg/dL)	10.0–40.0	-	43	34	<0.05
Complement4 (mg/dL)	0.00–8.00	-	9.42	67.95	NS
Hypersensitive C-reactive protein (mg/L)	0.00–0.05	-	0.04	0.09	<0.01
Procalcitonin (ng/ml)	0–40	-	36	46	NS
Brain natriuretic peptide (pg/ml)	7–40	-	13	17	NS
ALT (U/L)	13–35	-	20	19	NS
AST (U/L)	100.0–420.0	-	218	218	NS
Immune globulin (Ig)A (mg/dL)	860.0–1740.0	-	1330	1606	NS
Immune globulin (Ig)G (mg/dL)	30.0–220.0	-	115	60	NS
Immune globulin (Ig)M (mg/dL)	4.0–10.0*10^9	-	5.1	13	<0.05

Data are median, compared by the Mann–Whitney U-test between mild and severe COVID-19.

### Isolated RNA From Peripheral Blood Mononuclear Cells

Sequencing samples are organized in three sets of five, including five healthy donors, five mild COVID-19, and five severe COVID-19, with matched ages and genders and exclusion of patients with any comorbidity. PBMCs were isolated from peripheral venous blood by Ficoll density gradient centrifugation. Isolated cells were treated using RNAiso Plus (TAKARA) reagents under the instruction of the manufacturer for RNA isolation.

### Microarray Analysis

For preparations of rRNA depleted sequencing, mRNA was purified using the mRNA-ONLY™ Eukaryotic mRNA Isolation Kit (Epicenter). Then, fluorescent cRNA, which was amplified and transcribed from mRNA, was generated using the Arraystar Flash RNA Labeling Kit (Arraystar). A measure of 3 μg of purified cRNA per sample using the RNeasy Mini Kit (Qiagen) was used for hybridizations through Arraystar Human LncRNA Microarray V5.0 and then was washed, fixed, and scanned through the Agilent DNA Microarray Scanner (part number G2505C).

### Data Analysis

The gathered array images were analyzed using Agilent Feature Extraction software (version 11.0.1.1). The GeneSpring GX v12.1 software package (Agilent Technologies) normalized and processed the raw data. The flags from at least five out of 15 samples were positive in present or marginal (“All Targets Value”), and then the selected mRNAs were used for further analysis. The Benjamini corrected the *p*-values to control the false discovery rate (FDR). Differentially expressed genes (DEGs) conformed to the following criteria: adjusted *p*-value ≤ 0.05 and fold-change ≥ 2 between pairwise combinations of two groups. Also, DE lncRNAs (Raw ≥100) were further analyzed. The weighted gene co-expression network was constructed using the WGCNA package (R, Bioconductor), while the normalized expression index of genes (both mRNA and lncRNA) with top 30% standard deviation was chosen as an input. A gene tree was plotted to present the results of hierarchical clustering, and the dynamic tree-cutting algorithm was applied to segment the gene modules. Finally, the gene lists constituting each module were extracted for further analysis (Zhang and Horvath, 2005).

DE mRNAs, related to lncRNAs, were concluded based on the Pearson correlation coefficient (absolute value ≥ 0.9, *p*-value ≤ 0.01). Metascape Resource was applied for the GO and KEGG pathway annotations (https://metascape.org/gp/index.html). GO or KEGG pathways/terms with *p*-value < 0.01, a minimum count of 3, and an enrichment factor >1.5 were defined as a threshold, in which the term was statistically enriched. Heatmaps, ggplot2, correlation heatmaps, and bar plots were obtained by lc-bio.cn. online analysis. GO terms, Sankey diagrams, and principal component analysis (PCA) were obtained by http://www.bioinformatics.com.cn. CIBERSORT assessed the immune subtype, according to the expression file (https://cibersortx.stanford.edu/). ImmPort focused on immunologically relevant gene sets for analyzing cytokines and chemokines associated with lncRNAs (http://www.immport.org/).

### Construction of the Long Non-Coding RNA–mRNA Co-Expression Network

The lncRNA–mRNA network was constructed and visualized using Cytoscape (3.6.1) based on the Pearson correlation coefficient (absolute value ≥ 0.9, *p*-value ≤ 0.01). The protein–protein interaction (PPI) was established through the STRING database. The correlation between selected lncRNA and mRNA was analyzed using the co-expression network and Sankey diagrams (http://www.bioinformatics.com.cn).

### Treatment of HuT 78 Cells With Spike-ECD and Hemagglutinin Proteins

The human monocytic cell line THP-1 and the human T-cell line HuT 78 were purchased from the Cell Bank of Type Culture Collection of the Chinese Academy of Sciences. THP-1 was cultured in the RPMI 1640 medium, supplemented with 10% fetal bovine serum (Gibco), 1% penicillin/streptomycin, and 100 ng/ml phorbol 12-myristate 13-acetate (PMA; MCE, United States). After 24 h, THP-1 cells were stimulated by 50 ng/ml spike protein of severe acute respiratory syndrome coronavirus 2 (SARS-CoV-2) (GenScript) or the hemagglutinin (HA) protein of influenza A H1N1 (A/California/04/2009) (Sino Biological) for 24 h. The cells were then co-cultured with HuT 78 for another 6 and 12 h, followed by RNA isolation and the subsequent qRT-PCR assay.

### Quantitative RT-PCR

A measure of 1 μg of total RNA was reverse-transcribed to cDNA using HiScript II Q Select RT SuperMix (Vazyme). Quantitative real-time PCR was performed using SYBR qPCR Master Mix (Vazyme), and the amplifications were performed using QuantStudio 5 (Applied Biosystems). For quantification of gene expression, the 2-ΔΔCt method was used. The housekeeping gene glyceraldehyde-3-phosphate dehydrogenase (GAPDH) was used in all reactions as an endogenous control. The 5′–3′ sequences of primer pairs were as follows: GAPDH: GTCTCCTCT:GACTTC AAC AGCG (F) and ACCACCCTGTTGCTG TAG CCA A (R); AC005083.1: ACA​CCT​CTG​CCT​CTA​TGG​GA (F) and AGC​CAG​CCC​TTT​TGT​CTG​AT (R); AC136475.9: GGA​GTT​CCT​TCC​TTT​CGC​CT (F) and ATT​ACG​TCC​TGT​GCA​CGC​TC (R); XLOC_013290: GGG​TAT​CAG​GTG​TCT​GGG​TG (F) and CCA​GAA​ATC​CTG​CCA​CTC​CG (R); G004246: TGC​CAG​TAG​ACC​ATG​ACT​CG (F) and TTT​GGC​ACT​CTT​GGG​CAC​TT (R); CATG00000032642.1: AGC​ACT​TTG​CAG​AGG​GAC​AC (F) and ACA​GCA​ACA​AGG​GTT​GAG​GG (R); HLA-DRA: AGG​TTG​AGG​GAC​GGA​GAT​TT (F) and TGG​CTG​CAT​CTC​GAG​ACT​TT (R); HLA-DMA: GGG​AGA​TGC​TCC​TGC​CAT​TT (F) and AGC​GAT​AGG​AAA​CCC​TCT​GG (R); HLA-DMB: GAG​CTG​GCC​ACT​CTA​GTT​ACA (F) and GGA​GAG​GCA​TGG​TAG​CAT​CA (R); HLA-DQB2: CCGTGGAGTGGCGACCT (F) and GAC​ACA​GGC​AGC​TAG​GAA​TTC​TG (R); HLA-DOA: CAA​CGG​CCA​AAC​TGT​CAC​TG (F) and GTC​ATA​GAC​GTC​CTC​GGC​TG (R); LAT: GCAGACCCTTGGGGCCTA (F) and GCA​CAC​ACA​GTG​CCA​TCA​AC (R); VAV1: ACG​TCG​AGG​TCA​AGC​ACA​TT (F) and GGC​CTG​CTG​ATG​GTT​CTC​TT (R); LCK: GGA​GGA​CCA​TGT​GAA​TGG​GG (F) and CAT​TTC​GGA​TGA​GCA​GCG​TG (R).

### Statistical Analysis

GraphPad Prism 8.0 (GraphPad Software, San Diego, CA) was adopted for the t-test or Wilcoxon matched-pair test. Continuous variables were expressed as median (IQR) and compared using the Mann–Whitney U-test. Categorical measurement variables were displayed as count (%) and compared using the χ^2^ test or Fisher’s exact test. The Pearson correlation test was conducted to assess correlation between two quantitative variables. *p*-value < 0.05 was considered statistically significant.

## Results

### Sample Collection and Identification of Biomarker Long Non-Coding RNAs by Microarrays

All patients’ demographics, clinical characteristics, and laboratory results are shown in Tables 1, 2. The severe group exhibited lymphopenia, especially in T-cell cytopenia, and hyper-cytokines, notably increased TNF-α and IL-6 (Supplementary Figures S1A,B).

To discover the underlying mechanism of specific immune response in COVID-19 patients, PBMCs were isolated from laboratory-confirmed mild (*n* = 5) and severe COVID-19 patients (*n* = 5) as well as healthy controls (*n* = 5). There was no significant difference in age between each other (Supplementary Figure S1C).

**FIGURE 1:**
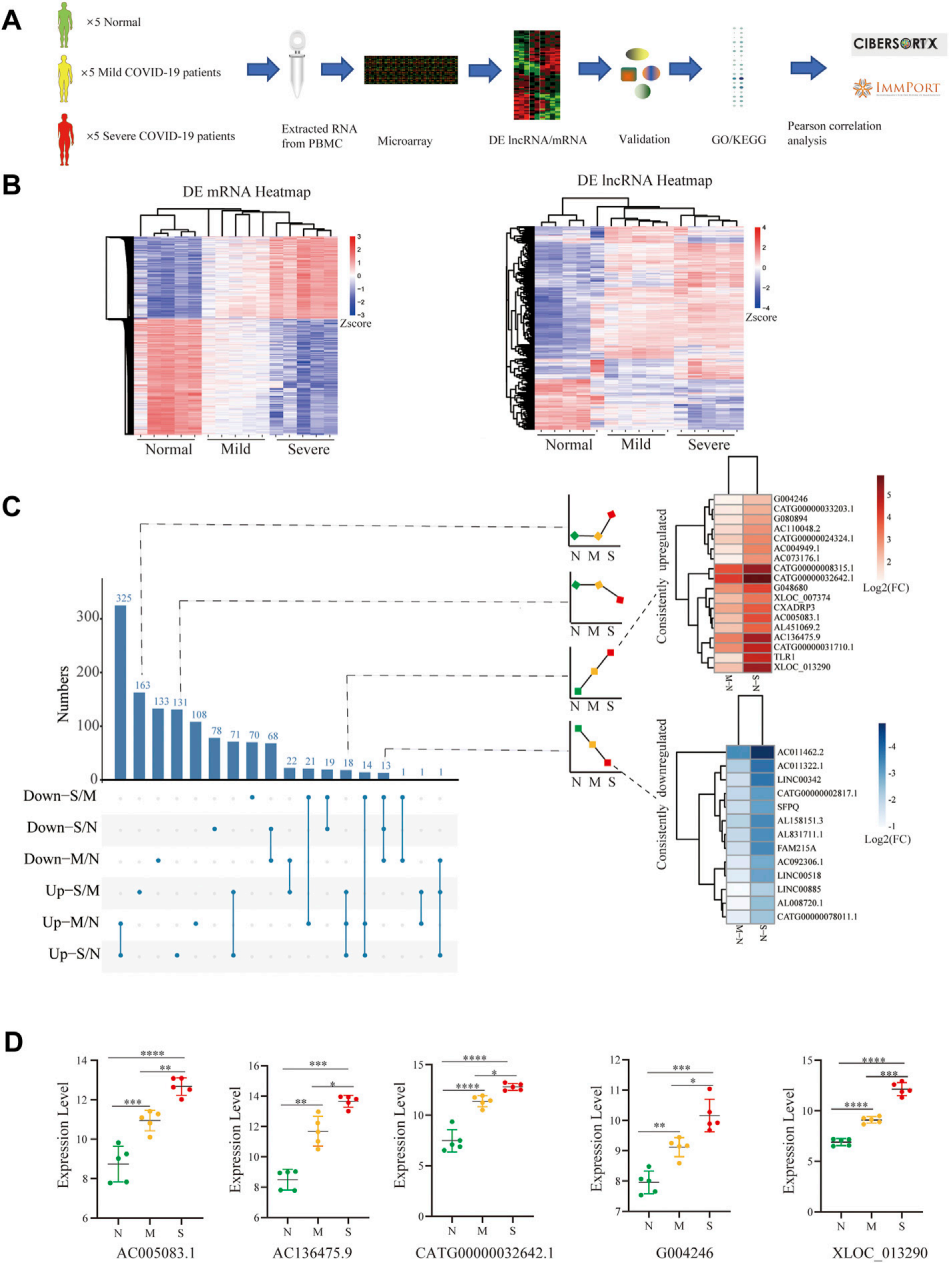
Identification of biomarker lncRNAs. **(A)** Research experimental design. **(B)** Heatmap comparison of DE lncRNAs and mRNAs within PBMCs from normal control (*n* = 5), mild (*n* = 5), and severe (*n* = 5) patients; each row and column respectively represent a separate gene and individual. **(C)** UpSet plot shows the number of DE lncRNAs with different tendencies; the heatmap shows the consistently increased or decreased lncRNAs by log2FC between mild vs. normal and severe vs. normal. **(D)** Filtered by *p*-value ≦ 0.01 and the length of lncRNAs among 400–3000 nts and the difference between pairwise combinations of two groups, with expression of lncRNAs in each group. N (Normal: *n* = 5), M (Mild: *n* = 5), and S (Severe: *n* = 5). Comparisons were done using the ANOVA test. Mild and severe patients were colored by yellow and red, respectively. Statistical comparisons are indicated by the arrows; ∗*p* < 0.05, **p < 0.01, ***p < 0.001, ****p < 0.0001; Mild and severe patients were colored by yellow and red, respectively.

Then microarray was applied and analyzed as the flow chart (Figure 1A). The corresponding expression profiles, in which each row represents a separate gene and each column represented a separate individual, were presented with clustered heatmaps, according to the severity of COVID-19 (Figure 1B). The volcano plots of DE lncRNAs (Raw ≥100) and DE mRNAs were shown by adjusted *p*-values (*p*-value < 0.05) and FC ratios (|log2FC|≥ 1) Supplementary Figure S2).

To screen out lncRNA signatures, UpSet analysis was performed in each comparison group (Figure 1C). Two types of lncRNAs were defined as candidate lncRNA biomarkers: 1) lncRNAs consistently downregulated (13) and 2) lncRNAs consistently upregulated (18). The lncRNAs were filtered by *p-*value ≦ 0.01 and the length of lncRNAs among 400–3000 nts and the difference between pairwise combinations of two groups, and only 5 lncRNAs conform to the criteria, including AC005083.1, AC136475.9, CATG00000032642.1, G004246, and XLOC_013290 (*p* < 0.001) (Figure 1D). These lncRNAs significantly increased in COVID-19, especially in severe COVID-19.

### Validation of Selected Long Non-Coding RNAs by Quantitative RT-PCR

**FIGURE 2:**
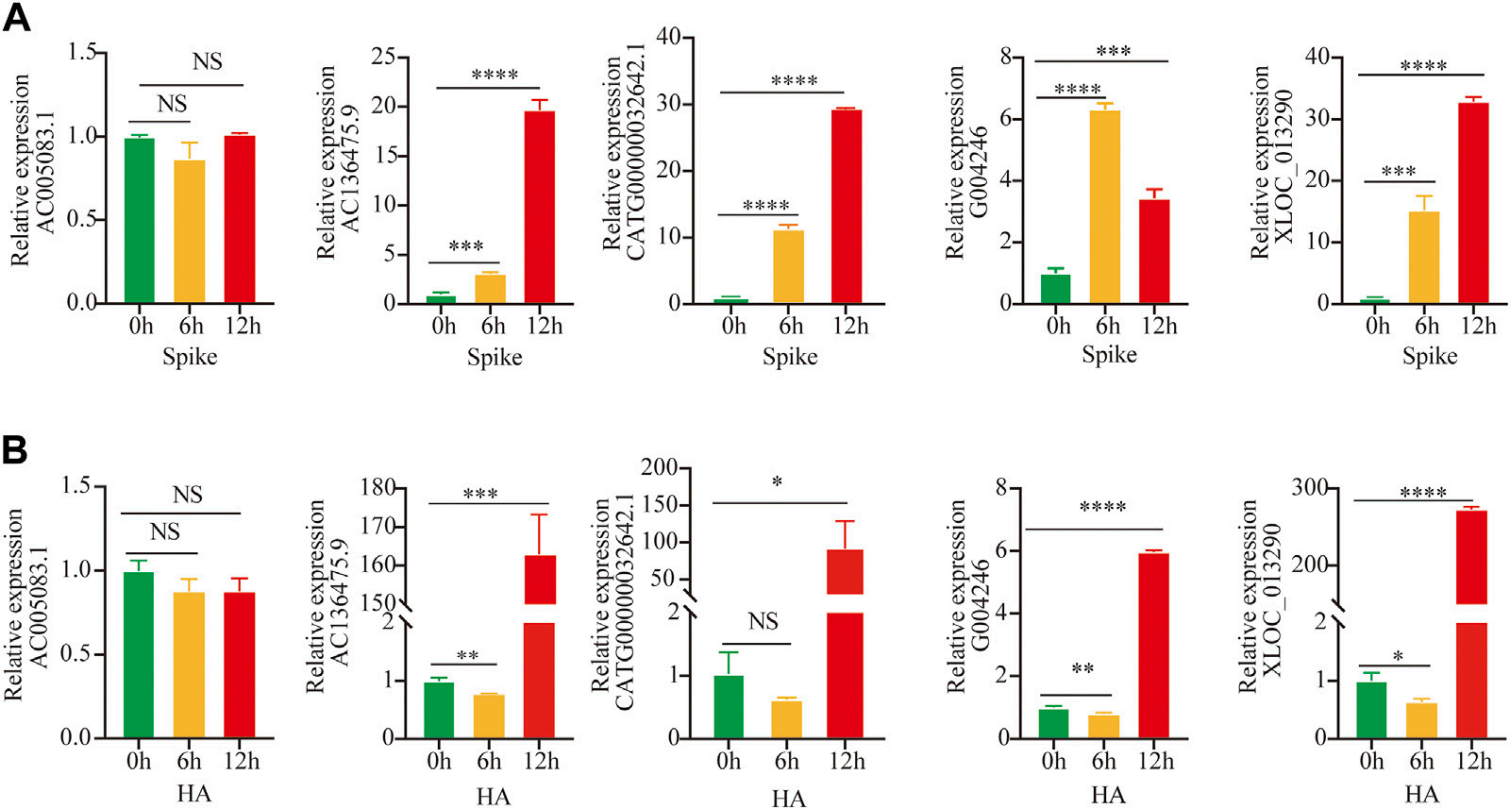
Validation by Quantitative RT-PCR. **(A,B)** Expressions of lncRNAs were detected after stimulation by S-protein and HA for 0, 6, and 12 h in HuT 78 cell co-cultured with the activated THP-1; statistical comparisons are indicated by the arrows; ∗*p* < 0.05, **p < 0.01, ***p < 0.001, ****p < 0.0001; comparisons were done using the ANOVA test.

For further validation of selected lncRNAs, we performed qRT-PCR. After stimulation with the S-protein and HA for 0, 6, and 12 h, we observed that four of five lncRNA, including AC136475.9, CATG00000032642.1, G004246, and XLOC_013290, significantly increased, excluding AC005083.1. (Figures 2A,B), consistent with the aforementioned microarray results. Also, the results suggested that the immune response to H1N1 and SARS-COV-2 was obviously different.

### Functional Enrichment With the Four Long Non-Coding RNAs in COVID-19

**FIGURE 3:**
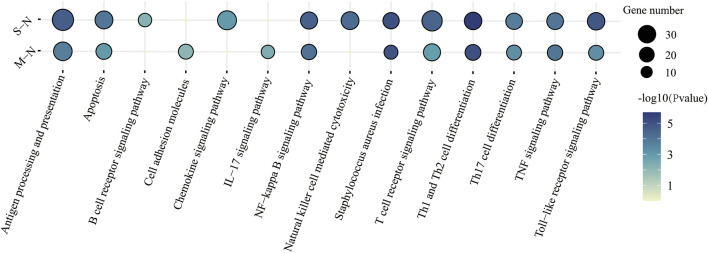
KEGG functional characteristics for the predicted target genes of four lncRNAs in immune response between mild and severe COVID-19.

We performed WGCNA analysis, in which selected lncRNAs played the role in viral release from the host cell. (Figure 3). To better understand the function of lncRNAs in COVID-19, we computed the Pearson correlation coefficient (PCC) between mRNAs and four lncRNAs. We identified 2,890 mRNAs correlated with lncRNA biomarkers (r > 0.9 and *p* ≤ 0.01) (Supplementary Table 2). Then, we performed GO and KEGG enrichment analysis (Supplementary Table 3).

The KEGG pathway was performed in Figure 3. “T-cell receptor signaling pathway,” “antigen processing and presentation,” and “apoptosis” were enriched in COVID-19, especially in severe patients. Surprisingly, *Staphylococcus aureus* infection was also found in COVID-19 patients. It suggested that patients were more susceptible to bacteremia, such as Gram-positive bacteria (Cusumano et al., 2020).

### Analysis of the Long Non-Coding RNA–mRNA Network in COVID-19

**FIGURE 4:**
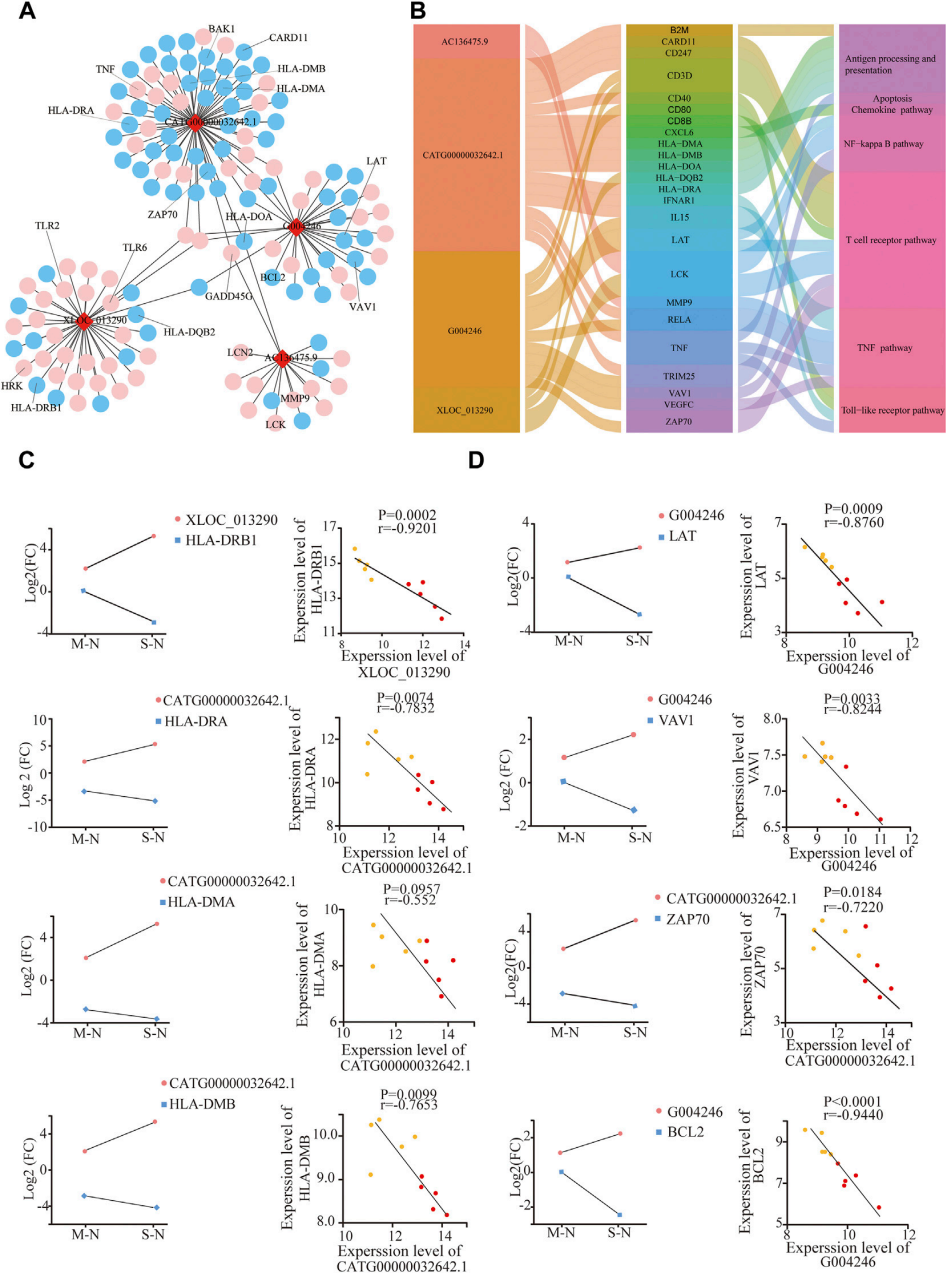
Analysis of the lncRNA–mRNA network in COVID-19. **(A)** Integrated lncRNA–mRNA network; **(B)** 4 lncRNA and selected 24 mRNA are shown using the Sankey diagram; **(C,D)** correlation between lncRNA and targeted mRNAs in mild and severe groups compared to healthy controls, respectively. The Y-axes correspond to the log2FC of lncRNAs and mRNAs. The number of samples: M (*n* = 5) and S (*n* = 5); mild and severe patients were colored by yellow and red dots, respectively.

Based on the aforementioned KEGG pathway, with the Pearson correlation (|R| > 0.94 and *p* ≤ 0.01), we constructed the lncRNA–mRNA co-expression network (Figure 4A). A total of 142 lncRNA–mRNA pairs were filtrated, and the top 24 genes were shown using the Sankey diagram (Figure 4B).

XLOC_013290 and CATG00000032642.1 were negatively correlated with HLA-DQB2 and HLA-DRB1, respectively (Figure 4C). Also, HLA-DRA, HL-DMA, and HL-DMB decreased in COVID-19, especially in severe cases, consistent with Mudd PA’s result, who reported that abundances of HLA-DR of monocytes significantly reduced in COVID-19 (Mudd et al., 2020). After stimulation with the S-protein for 0, 6, and 12 h *in vitro*, the mRNA HLA-DOA-related antigen presentation appeared to have a decline in trend after an initial increase (Supplementary Figure S4A).

LAT, VAV1, ZAP70, and CARD11, as the key components of T-cell receptor, exhibited a significant decrease with severity in COVID-19, negative with G004246 and CATG00000032642.1. (Figure 4D). Meanwhile, the gene expression of VAV1 and LCK also declined, after stimulation with the S-protein for 12 h *in vitro* (Supplementary Figure S4B). Moreover, BCL2, an important antiapoptotic protein, shows a decreasing trend in COVID-19. Also, GADD45G, HRK, and BAK, as proapoptotic proteins, showed a significant increase with severity in COVID-19 (Figure 4D, Supplementary Figure S4C). These results indicated that SARS-CoV2 could induce T-cell apoptosis. Also, higher levels of both TLR2 and TLR6 genes were observed with the severity of COVID-19 (Supplementary Figure S4D) (Taniguchi-Ponciano et al., 2021). These results implied that lncRNAs could participate in T-cell reduction, with the dysfunction of TCR and HLA as well as increased apoptotic proteins.

### Validation of Long Non-Coding RNAs as Biomarkers by Differences in Immune Cell Compositions, Especially in T-Cell Cytopenia

**FIGURE 5:**
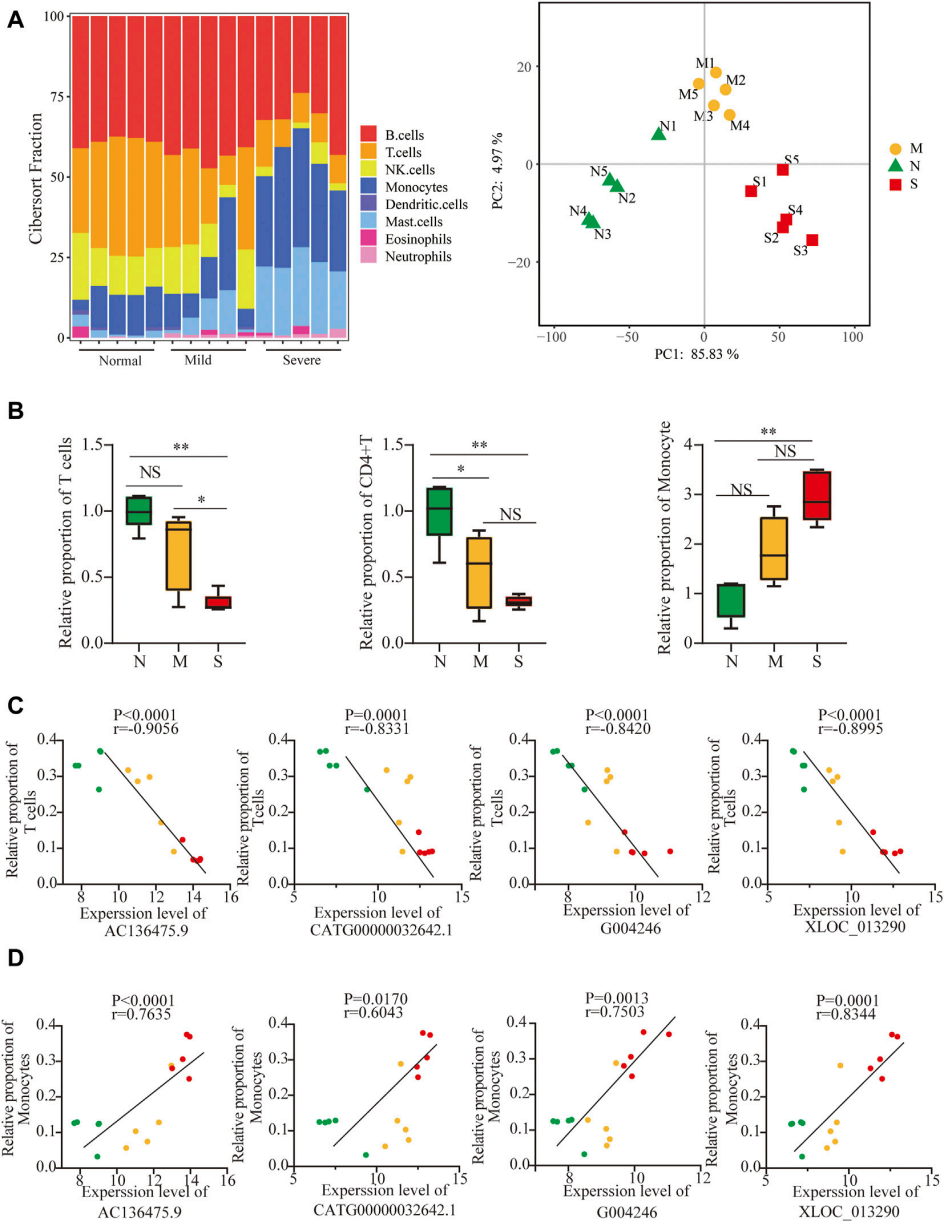
Validation of lncRNAs as biomarkers by differences in immune cell compositions. **(A)** Profiles of immune subtypes are shown using bar plots; PCA between normal and COVID-19. **(B)** Difference in immune subtypes among three groups is shown using the Mann–Whitney test, including T cells, CD4+ T cells, and monocytes. **(C, D)** Correlation between 4 lncRNAs and T cells and monocytes in normal, mild, and severe groups. The normal, mild, and severe patients were colored by green, yellow, and red dots, respectively. ns: non-significant; *p < 0.05; **p < 0.01.

Next, to further identify whether lncRNAs play a role in T-cell cytopenia under COVID-19 conditions, the potential mRNAs of lncRNAs were applied with CIBERSORT and eight major cell subpopulations were successfully identified in peripheral blood using the bar plot. (Figure 5A). Principal component analysis (PCA) showed that there were significant differences in immune cell composition, also in the severity of COVID-19 (Figure 5A).

The fraction of total T cells was lower in COVID-19 (Figure 5B), especially in CD4T, and not in CD8T (Supplementary Figure S5A), and only marginal differences in B cells were observed (Supplementary Figure S5A). The adaptive immune cell subsets were depleted in COVID-19, including follicular helper T cells (TFh), regulatory T cells (Tregs) cells, and gamma delta T (γδT) cells (Supplementary Figure S5B). Surprisingly, the proportion of monocytes significantly increased in COVID-19 (Figure 5B). In contrast, the frequencies of activated natural killer (NK) cells and dendritic cells (DCs) decreased in both mild and severe COVID-19 (Supplementary Figure S5C). The frequency of neutrophils increased slightly in COVID-19, although the neutrophils were normally excluded from PBMCs (Supplementary Figure S5C). Furthermore, 4 lncRNAs were negatively correlated with T-cell cytopenia but positively correlated with an increased frequency of monocytes (Figures 5C,D). It implied that 4 lncRNAs might participate in not only T-cell cytopenia but also increased monocytes.

Monocytes have been implicated in the physiopathology of COVID-19, and differences in transcriptional signatures about monocytes are shown in Supplementary Figure S6A based on Aaron J. Wilk’s single-cell RNA sequencing (scRNA-seq) (Wilk et al., 2020). Both Mudd et al. (2020) and Zhou et al. (2021) reported SARS-CoV2 could inhibit antigen-presenting ability in monocytes, which led to T-cell cytopenia.

Furthermore, both Wen et al. (2020) and Zhou F et al. (2020) observed that monocytes significantly increased and proved that pro-inflammatory cytokines and chemokines genes were also upregulated. A distinct change in cytokines and chemokines was shown in mild and severe COVID-19, derived from ImmPort (Bhattacharya et al., 2018), which were associated with lncRNAs (|R| > 0.90 and *p* ≤ 0.01). (Supplementary Figure S6B,C).

It indicated that SARS-Cov2 led to the upregulation of lncRNA, T-cell cytopenia, and transcriptional differences of monocytes, with a distinct change in cytokines and chemokines, especially in severe COVID-19.

## Discussion

Previous studies reported lncRNAs played a potential role in immune response. In order to find the lncRNA associated with COVID-19, we performed a whole genome microarray of PBMCs from COVID-19 and healthy donors.

In this study, we have demonstrated that 1) lncRNAs AC136475.9, CATG00000032642.1, G004246, and XLOC_013290 are active participants in COVID-19; 2) their function enriched in dysregulation of TCR and HLA as well as increased apoptotic proteins, which could be in part of T-cell cytopenia; and 3) they also participated in monocyte reprogramming, with a distinct change in cytokines and chemokines.

Both the routine laboratory results and bioinformatics analysis demonstrated that lymphopenia in the periphery was the prominent feature in COVID-19, which was similar to recently published data (Giamarellos-Bourboulis et al., 2020; Huang et al., 2020; Tan et al., 2020; Chen et al., 2020) (Supplementary Figure S1). Also, lymphocyte counts in severe cases performed lower than those in mild cases, which implied that the degree of lymphocytopenia was relevant to the severity of COVID-19 (Qin et al., 2020; Wang et al., 2020). Moreover, our results showed that the absolute number or frequency of several immune subsets in severe COVID-19 significantly decreased compared to that in mild COVID-19, including CD4 T cells, CD8 T cells, and natural killer cells (Kuri-Cervantes et al., 2020) (Supplementary Figure S1, Figure 5).

Several explanations accounted for lymphopenia, caused by SARS-CoV-2. First, the peripheral lymphopenia is possibly attributed to the redistribution or recruitment of lymphocytes from the periphery to the respiratory tract or lymphoid organs, accompanied by the accumulation of lymphocytes (Liao et al., 2020; Wichmann et al., 2020). Second, enrichment analysis included the “apoptosis signaling pathway,” and viral infection of lymphocytes might lead to cell death by apoptosis, necrosis, or pyroptosis (Yue et al., 2018; Tan et al., 2007). Last, increased levels of cytokines may be associated with lymphopenia. TNF-а led to lymphocyte apoptosis, particularly for CD8 T-cell apoptosis (Mehta et al., 2018). IL-10 was known as an immunosuppressive effect, which also induced CD4 T and CD8 T inactivation (Brooks et al., 2006). Type-I IFN had an impact on lymphocyte proliferation, apoptosis, and expression of cytokines and cytokine receptors and disrupted modulation of S1P-S1P1 for lymphocyte recirculation (Kamphuis et al., 2006). Moreover, COVID-19 patients who were admitted had a substantial accumulation of inflammatory cytokines, including TNF-а, IL-6, IL-2, and IL-10 (Supplementary Figure S1B), especially in severe COVID-19.

Meanwhile, increased plasma inflammatory biomarkers were accompanied by worsened symptoms, including C-reactive protein, ferritin, and D-dimers, and neutrophil-to-lymphocyte ratio (Wu et al., 2020; Ruan et al., 2020; Zhou et al.,2020). Although the fraction of T cells decreased in severe COVID-19, the frequency of monocytes and the levels of inflammatory cytokines and chemokines evidently increased (Chen et al., 2020; Gong et al., 2020; Huang et al., 2020; Qin et al., 2020; Yang L et al., 2020). The aforementioned results indicated there is a potential interaction between T-cell reduction and an increased proportion in monocytes.

Aaron J. Wilk found that the phenotype of the monocyte was remodeled, in which the CD16^+^ monocyte was depleted, but they did not find the series of pro-inflammatory cytokine genes coding in the monocyte (Wilk et al., 2020). A single-cell analysis of PBMCs from COVID-19 found that CD14^+^ monocytes were significantly increased, while an observed decrease was shown in CD16^+^ monocytes (Wen et al., 2020; Huang et al., 2021). Zhou Y et al. (2020) not only observed that CD14^+^CD16^+^ monocytes significantly increased but also proved that monocytes secreted a large amount of IL-6 and GM-CSF. Moreover, substantial pro-inflammatory cytokines and chemokines genes, associated with CD14^+^ monocytes, were upregulated, such as IL1β, JUN, and CCL4 (Wen et al., 2020). In addition, they suggested that pro-inflammatory monocytes contribute to the potential cytokine storm and also implied that various cytokines and chemokines migrate to lung and lymph organs and then activate a series of inflammatory immune cells and aggravate lung injury.

Despite the extensive clinical research studies on lncRNAs, several questions still need to be further addressed. Several risk factors, which affected the expression of peripheral lncRNAs, must be observed to make more accurate predictions of potential lncRNAs. First, Pujadas et al. (2020) reported that there is an independent relationship between high viral load and mortality. Among the enrolled patients in isolation wards or intensive care units, the absolute viral load was not measured. The laboratory result of SARS-COV-2 was positive or not. It is difficult to determine the accuracy of lncRNAs in the development and progression of COVID-19. Second, recent research indicated that some of the risk factors could alter the potential lncRNA expression in peripheral immune response, including age (Lee et al., 2012), sex (Scully et al., 2020), and some other comorbidities. In our studies, the majority of the samples were male. As a specific biomarker in COVID-19, whether lncRNAs were applied to female or not, it is needed to be further verified. Furthermore, as the positive control, patients, who did not suffer from COVID-19 but from pneumonia or acute respiratory distress syndrome, were not enrolled, and the specificity of non-coding RNA biomarkers still need validation.

## Conclusion

In this study, we reported the potential lncRNAs and their correlation with T-cell cytopenia and an increased frequency of monocytes in COVID-19. Our analysis provided bases for therapeutic targets in COVID-19 as well as the SARS-CoV-2-specific vaccines.

## Data Availability

The datasets presented in this study can be found in online repositories. The names of the repository/repositories and accession number(s) can be found in the article/Supplementary Material.

## Supplementary Material

Supplementary Figure S1 Difference of peripheral lymphocyte subsets and cytokines in COVID-19 patients. **(A)** Cell counts or frequency of lymphocytes, neutrophils, monocytes, CD3+ T cells, CD4+ T cells, CD8+ T cells, NK cells, and B cells in mild and severe groups were analyzed. **(B)** Levels of IL-6, TNF-a, IFN-γ, IL-2, and IL-10 from mild and severe patients. **(C)** Characteristics of all subjects for the microarray. The shaded region shows the normal range of the indicated cell subset. M: mild; S: severe; Mild and severe patients were colored by yellow and red, respectively. The data were compared by the Mann–Whitney U-test. ns: non-significant; ∗p < 0.05, **p < 0.01, ***p < 0.001, ****p < 0.0001.

Supplementary Figure S2 Volcano plot of DE lncRNAs and mRNAs among three groups.

Supplementary Figure S3 WGCNA was performed for analysis of co-relation between lncRNA and mRNA. The brown and green modules respectively are AC136475.9, G004246, XLOC_013290, and CATG00000032642.1.

Supplementary Figure S4 Expression and correlation of lncRNAs and mRNA; **(A, B)** Expression of mRNA, related antigen presentation, and TCR were detected by RT-PCR after stimulation by S-protein and HA for 0, 6, and 12 hours in HuT 78 cells co-cultured with the activated THP-1; **(C)** correlation between lncRNAs and apoptosis; **(D)** correlation between lncRNAs and TLRs. Statistical comparisons are indicated by the arrows; ns: non-significant; ∗p < 0.05, **p < 0.01; Comparisons were done by the ANOVA test.

Supplementary Figure S5 Difference in immune cells among three groups; the normal, mild, and severe patients were colored by green, yellow, and red, respectively. Data were compared by the Mann–Whitney test; ns: non-significant; *p < 0.05; **p < 0.01.

Supplementary Figure S6 Cytokine and chemokine profiles in COVID-19 patients. **(A)** Heatmap of DE genes related to monocytes; **(B)** number of genes in 17 immune-related pathways; **(C)** heatmap comparison of chemokines and cytokines; **(D)** correlation heatmap between 4 lncRNAs with cytokines and chemokines. *p*-values generated by one-way ANOVA with multiple comparisons by Tukey’s test. ns: non-significant; *p < 0.05; **p < 0.01, ***p < 0.001, ****p < 0.0001.
