# Supplementary figures and images for: Comparative Analysis of Long Non-Coding RNA Expression and Immune Response in Mild and Severe COVID-19
Source: Front Mol Biosci. 2022 Apr 27;9:835590. doi: 10.3389/fmolb.2022.835590 (PMC9094366; doi:10.3389/fmolb.2022.835590)

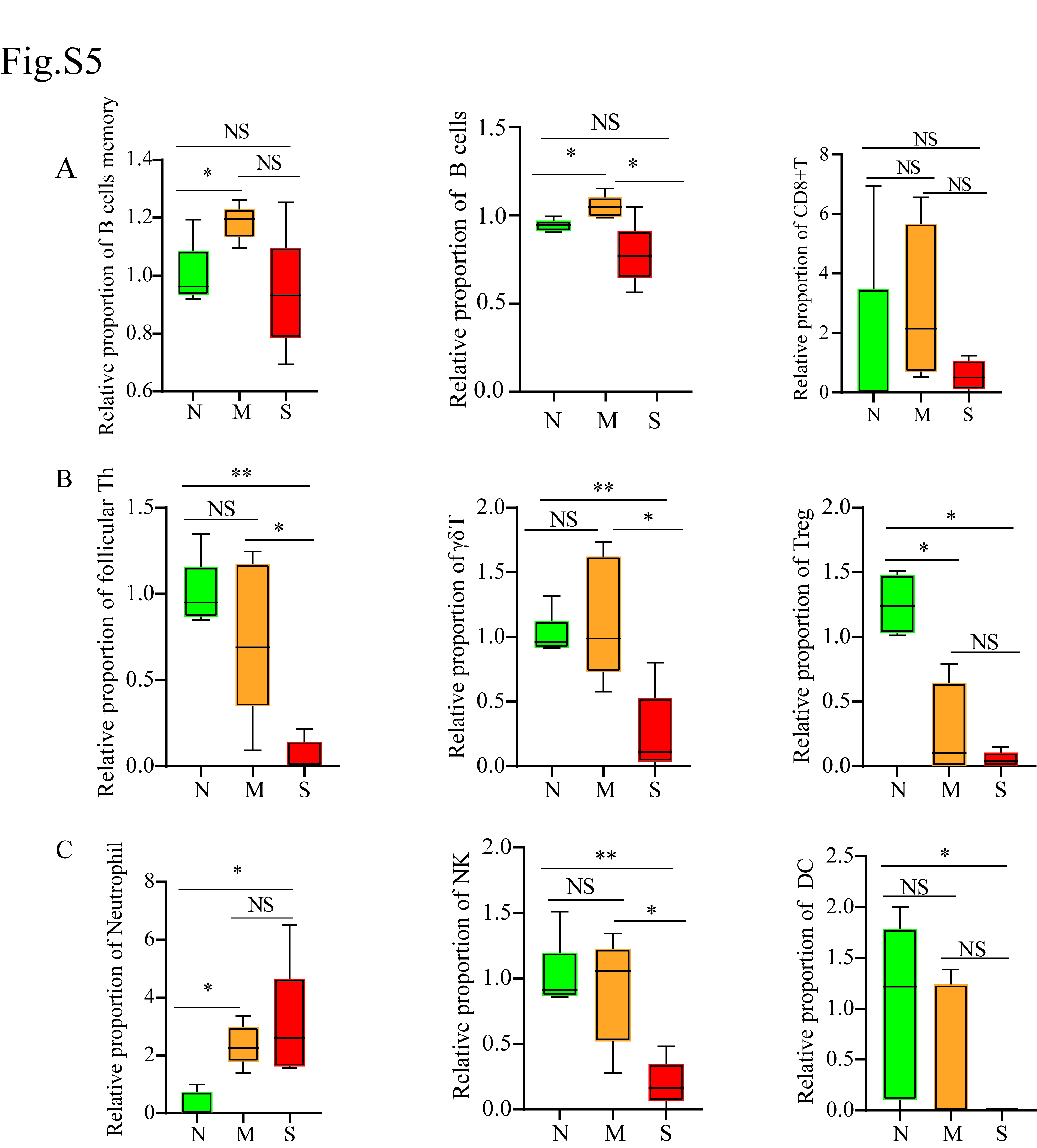

Supplement: Supplementary file 4 [file Image4.TIF]
